# The Natural Statistics of Audiovisual Speech

**DOI:** 10.1371/journal.pcbi.1000436

**Published:** 2009-07-17

**Authors:** Chandramouli Chandrasekaran, Andrea Trubanova, Sébastien Stillittano, Alice Caplier, Asif A. Ghazanfar

**Affiliations:** 1 Neuroscience Institute, Princeton University, Princeton, New Jersey, United States of America; 2 Department of Psychology, Princeton University, Princeton, New Jersey, United States of America; 3 GIPSA Lab, Grenoble, France; 4 Department of Ecology & Evolutionary Biology, Princeton University, Princeton, New Jersey, United States of America; University College London, United Kingdom

## Abstract

Humans, like other animals, are exposed to a continuous stream of signals, which are dynamic, multimodal, extended, and time varying in nature. This complex input space must be transduced and sampled by our sensory systems and transmitted to the brain where it can guide the selection of appropriate actions. To simplify this process, it's been suggested that the brain exploits statistical regularities in the stimulus space. Tests of this idea have largely been confined to unimodal signals and natural scenes. One important class of multisensory signals for which a quantitative input space characterization is unavailable is human speech. We do not understand what signals our brain has to actively piece together from an audiovisual speech stream to arrive at a percept versus what is already embedded in the signal structure of the stream itself. In essence, we do not have a clear understanding of the natural statistics of audiovisual speech. In the present study, we identified the following major statistical features of audiovisual speech. First, we observed robust correlations and close temporal correspondence between the area of the mouth opening and the acoustic envelope. Second, we found the strongest correlation between the area of the mouth opening and vocal tract resonances. Third, we observed that both area of the mouth opening and the voice envelope are temporally modulated in the 2–7 Hz frequency range. Finally, we show that the timing of mouth movements relative to the onset of the voice is consistently between 100 and 300 ms. We interpret these data in the context of recent neural theories of speech which suggest that speech communication is a reciprocally coupled, multisensory event, whereby the outputs of the signaler are matched to the neural processes of the receiver.

## Author Summary

When we watch someone speak, how much work is our brain actually doing? How much of this work is facilitated by the structure of speech itself? Our work shows that not only are the visual and auditory components of speech tightly locked (obviating the need for the brain to actively bind such information), this temporal coordination also has a distinct rhythm that is between 2 and 7 Hz. Furthermore, during speech production, the onset of the voice occurs with a delay of between 100 and 300 ms relative to the initial, visible movements of the mouth. These temporal parameters of audiovisual speech are intriguing because they match known properties of neuronal oscillations in the auditory cortex. Thus, given what we already know about the neural processing of speech, the natural features of audiovisual speech signals seem to be optimally structured for their interactions with ongoing brain rhythms in receivers.

## Introduction

Organisms are exposed to a continuous stream of signals which are dynamic, multimodal, extended, time varying in nature and typically characterized by sequences of inputs with particular time constants, durations, repetition rates, etc. [1]. This complex input space is transduced and sampled by the respective sensory systems and transmitted to the brains of the organisms where they modulate both neural activity and behavior over multiple time scales [2]. The shaping of brain activity and behavior by the environment has long been noted. Barlow [3], for example, suggested that exploiting statistical regularities in the stimulus space may be evolutionarily adaptive. According to this approach, sensory processing would encode incoming sensory information in the most efficient form possible by exploiting the redundancies and correlation structure of the input. In essence, neural systems should be optimized to process the statistical structure of sensory signals that they encounter most often [4]. Often overlooked in these studies is recognition that an organism's experience of the world is profoundly multisensory, and it is likely that multiple overlapping and time-locked sensory systems enable it to perceive events and interact with the world [5].

One class of multisensory signals for which a careful input space characterization is unavailable is human speech. To be sure, there are numerous acoustic analyses of human speech, but there are relatively few for *audiovisual* speech. Multisensory speech is the *primary* mode of speech perception and it is not a capacity that is “piggybacked” on to auditory speech perception [6]. Human speech is bimodal, dynamic and forms a large proportion of the sensory signals encountered by humans in social contexts. Although a remarkable amount of research effort has been expended on the psychophysics and neural basis of audiovisual speech (reviewed in [7]), a mechanistic account of audiovisual speech processing remains elusive. Several issues, such as the type of information extracted from each modality, static versus dynamic features, and the stage of integration for visual and auditory inputs are unresolved [6],[7]. We do not know what features the brain has to actively extract and combine from the audiovisual speech stream to arrive at a percept versus what is already embedded in the signal structure of the stream itself. In essence, we do not have a clear understanding of the natural statistics of audiovisual speech.

In this study, we examine the natural statistics of audiovisual speech in a framework similar to prior studies of natural visual scenes [8] and auditory soundscapes [9]. We analyzed speech from three different databases comprised of two different languages, meaningful versus nonsensical sentences, and staged versus conversational speech. Our analyses were conducted using methods developed to analyze neurophysiological data [10]. The analysis was motivated by recent neural theories of speech and audiovisual speech perception [11],[12].

We identified the following major statistical features of audiovisual speech. First, we observed robust correlations and close temporal correspondence between the area of the mouth opening (or inter-lip distance) and the auditory envelope [13]. Second, we found that the strongest correlation between the area of the mouth opening and vocal acoustics occurred in two bands–below 1 kHz and between 2 and 3 kHz—the latter corresponding to formants (or vocal tract resonances). Third, we observe that *both facial movements and voice envelopes* are temporally modulated in the 2–7 Hz frequency range, which overlaps with the timescale of the syllable [11],[14]. This result was consistent across two languages and different speech contexts. Finally, we show that the timing of mouth movements relative to the onset of the voice is consistently between 100 and 300 ms. We suggest that these statistical properties of audiovisual speech agrees are consistent with ideas suggesting that speech is inherently amodal [15] and are well matched to the properties of neural circuits involved in the processing of speech signals.

## Methods

### Databases

Our measurements were performed on two different audiovisual databases and an x-ray database of natural speech to ensure that our conclusions were not constrained by the methodology, language or the context and length of the speech segments. One audiovisual database was in British English, the other consisted of conversational French. The x-ray database was in American English. Descriptions of the databases and their features are provided below.

#### GRID corpus

The bulk of our analyses used the GRID corpus, a large multi-talker audiovisual sentence corpus in British English with high quality audio and video recordings [16]. The corpus consists of 34 speakers each producing 1000 sentences, and each sentence in this database contained six words including a command, color, preposition, letter, digit, and adverb. All the categories had four options except for the digit, which included numbers from zero to nine and the letter category which included all letters in the alphabet expect for ‘W’. Example sentences produced by a speaker in this database were “bin blue at A1 again” or “place green by D2 now”. Speakers produced such sentences at a normal speaking rate and were asked to complete the sentence in 3 seconds. Video was digitized at 25 frames per second, which provided us with an estimate of the lip parameter once every 40 milliseconds. Our lip tracking algorithm did not perform equally well for all speakers nor all sentences within a speaker. We therefore only chose the sentences where the tracking was robust throughout the duration of the sentence. This meant that we had a variable number of sentences on a per subject basis. The data we report for the GRID corpus was from 20 subjects. Specifically, we analyzed 50 sentences each for 11 subjects and 25 sentences each for nine other subjects for a total of 775 sentences.

#### Wisconsin x-ray microbeam database

The second database we used was the American English X-ray microbeam database from the University of Wisconsin. This corpus consisted of a variety of materials such as the DARPA TIMIT (Texas Instruments – Massachusetts Institute of Technology) sentences [17], small words, or long passages of prose. We analyzed five segments of connected prose to assess the properties of speech over longer segments than the GRID corpus allowed. Another major advantage of this database was its higher sampling rate. The lip markers were sampled at 146 Hz, allowing us to provide robust estimates of lip movements *before* the onset of the vocal sound. This database was constructed by asking speakers to repeat materials presented to them and did not contain any casual speech. Further details of the data acquisition can be found in [18].

#### French spontaneous speech database

The final database we considered was a French database based on an unstructured conversation between two native French speakers. This database was used to develop a method to detect voice-related activity using the relationships between lip activity and the sound [19]. The two speakers recorded in this database were assigned a variety of tasks such as providing answers to a word game, finding solutions to riddles or playing other language games. This resulted in a database of natural audiovisual speech interactions. As far as we know, this is the only available database consisting of casual or conversational audiovisual speech.

### Signal extraction

All analyses were carried out in MATLAB (Mathworks, Natick, USA) using a mixture of custom built scripts and native functions.

#### Extracting the visual signal

**Figure 1:**
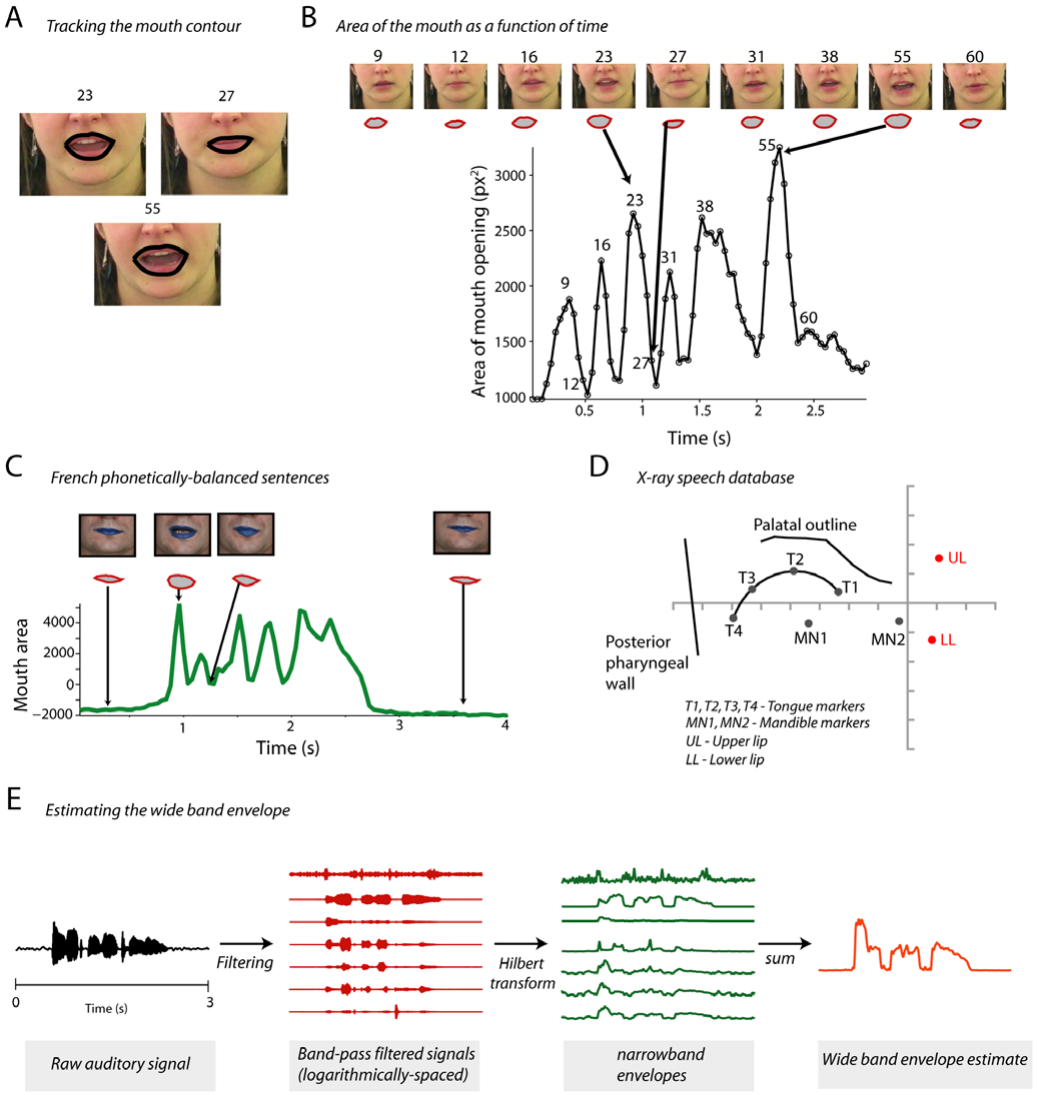
Analysis framework for the visual and auditory components of natural speech. A - Shows the frames from the orofacial region of the mouth for the sentence “Bin red by y2 now” spoken by a single female speaker in the GRID corpus. Black lines denote the fitted lip contour using our contour fitting algorithm (see methods). B - Top panel, shows the frames and the corresponding contours for several frames for the same sentence from the same speaker shown in A. The bottom panel shows the estimated area for each mouth contour as a function of time. X-axes depict time in seconds; y-axes depict the area of the mouth opening in pixel squared. Arrows point to specific frames in the time series depicting different amounts of mouth opening. C - Similar analysis of the French phonetically balanced sentences. Tracking in this case was facilitated by the blue lipstick applied to the lips of the speaker, which allowed for automatic segmentation of the lips from the face. D - A sagittal frame from the x-ray database with the pellet positions marked. Eight different pellets were recorded for this database of human speech. We analyzed the inter-lip distance between the markers UL (upper lip) and LL (lower lip). E - Estimation procedure for the wideband envelope. The signal is first band pass filtered into narrow bands according to a cochlear frequency map. The narrowband envelopes are obtained by taking the Hilbert transform and then computing the absolute value. The wideband envelope is estimated as the sum of the narrowband envelopes.

For the GRID audiovisual database, we used a computer vision approach to track the lip contours in the video on a frame-by-frame basis [20],[21]. The area of this lip contour provided an estimate of the area of the mouth opening. Figure 1A shows an example analysis of three frames of a sentence ‘bin red by Y2 now’ uttered by a female speaker. Figure 1B shows several such frames of the sentence and the corresponding estimate of the mouth area as a function of time for the same sentence. The area of the mouth opening in pixels provides us with a frame-by-frame estimate of the mouth area. The area of the mouth opening changes as a function of time and we refer to it as the mouth area function. This metric is correlated with other measures such as the inter-lip distance [13] and is therefore an excellent measure of the visual content of audiovisual speech.

Figure 1C shows the frames and the corresponding extracted lip area for the French audiovisual database. Estimating the visual parameters in the French audiovisual database was considerably easier since the subjects wore blue lipstick to help capture the signal using the ICP “face processing system”. This method uses thresholding with the chroma key system and contour tracking algorithms to track the lips on a frame-by-frame basis [22]. Relevant visual parameters such as the lip width, lip height, and area of the mouth opening were extracted and used for subsequent analysis.

For the x-ray database, we considered the distance between the *y* coordinates of the upper lip (UL) and lower lip (LL) as markers for the mouth opening and hereafter refer to it as the inter-lip distance (Figure 1D).

#### Measuring the auditory envelope

We estimated the wideband envelope by adapting a previously devised method using band-passed filters and the Hilbert transform [23]. Figure 1E shows the steps involved in the analysis of the auditory signal. The raw speech waveform was first band-pass filtered into different frequency bands with cut-off frequencies so that the bands spanned equal widths on the cochlear map. The absolute value of the Hilbert transform of a band-passed signal gives an estimate of the envelope for that frequency band and each of these is termed a ‘narrowband envelope’ [23],[24]. We summed these individual narrowband envelopes to arrive at the wideband envelope. Both narrow and wideband envelopes were used in subsequent analyses. The cut-off frequencies were identified using the MATLAB subroutine provided by Bertrand Delgutte and others (http://research.meei.harvard.edu/chimera/More.html) [23]. The cutoff frequencies in Hz were 100, 128, 161, 197, 238, …1583, 1798, 2040, 2313, 2621,…3795, 4289, …6175, 6969, …8868, 10000. Filtering was performed by using a two-way least-squares filter between two successive cutoff frequencies. Care was taken to ensure that the overlap of the pass-bands of the filters were minimal, although some overlap was inevitable. Such close spacing was also chosen between frequencies to ensure that we sampled the frequency space as much as possible in order to identify the spectral regions which best correlated with the area of the mouth opening. Since the mouth area function was sampled at 25 Hz, we re-sampled the auditory envelope at 25 Hz to allow us to correlate the visual and auditory signals. Different upper cut-offs were chosen for the three databases due to differences in sampling rates. The sampling rates and the corresponding filter bank ranges were 50 KHz (100 Hz–10 KHz) for the GRID corpus, 21.73 KHz (100 Hz–8 KHz) for the Wisconsin x-ray database, and 44.1 KHz (100 Hz–10 KHz) for the French database.

### Correlation analysis

A Pearson correlation analysis was performed to relate the visual components of speech as indexed by the mouth area function/inter-lip distance and the auditory component, which could be either wideband or narrowband envelopes. As outlined in the previous section, the envelope was first computed and then re-sampled to 25 Hz to ensure that both auditory and visual signals shared the same sampling rate. Since such correlations can arise purely due to chance, we performed a shuffled correlation analysis on a subject-by-subject basis. Shuffled correlations were computed by correlating the mouth area function from one sentence with the auditory envelopes from all other sentences produced by that particular subject. This provided a rigorous test to ensure that the correlations were not due to arbitrary covariation between two positive signals. In the case of the Wisconsin x-ray database, speech lengths were not consistent across multiple passages. We therefore chose the function (inter-lip distance or the envelope), which was smaller in length for the two modalities and performed subsequent shuffled correlations.

### Spectral analysis of auditory and visual speech signals

Since we were interested in how audiovisual speech signals may relate structurally to neural signals, we measured them using the same approaches and spectral analysis techniques used in the neurophysiology literature [25]–[28]. We describe these here.

To estimate temporal frequency modulations, we used multitaper Fourier methods (Chronux toolbox, www.chronux.org) to derive the spectra for the visual (mouth area and the inter-lip distance) and auditory signals. Since natural signals and sound typically display a 1/f spectrum, we fitted a function of the form *Y = Af ^−α^*. Deviations from the 1/f fit suggest certain rhythmic aspects of the signal. The parameter *A* is a scaling parameter and alpha is the exponent. The analysis was performed on the logarithmic version of the same equation, i.e. *logY = logA−α logf*. This allowed for linear methods and avoided nonlinear fitting. Corresponding R^2^ values and parameters are reported.

We used coherence to estimate the relationship between the visual and auditory signal as a function of modulation frequency. Coherence is a metric, which estimates the relationships between two time series as a function of frequency and does not have normalization problems [10],[29]. We estimated coherence between auditory spectral frequency bands and the visual signals (mouth area or inter-lip distance). The coherence was estimated as follows. For each sentence from each subject, we computed the coherence between the narrowband envelope and the mouth area function (or the inter-lip distance). This provided, for each spectral frequency band, an estimate of the coherence between the auditory signal in that frequency band and the visual signal. Computing across all the spectral frequency bands gave us an estimate of the coherence for each sentence as a function of both spectral and temporal frequency. We then averaged this coherence estimate across all sentences for each subject and then subsequently across subjects.

### Estimating time-to-voice for bilabial and labiodental consonants

The time delay between the onset of mouth movements and onset of the voice component is the ‘time-to-voice’. It is shaping up to be an important feature in determining audiovisual interactions in the neocortex [25],[30] and an important part of neural theories of audiovisual speech [12]. All time-to-voice measurements were performed using the Wisconsin x-ray database because of its high 146 Hz sampling rate, which allowed us to get smooth lip motion trajectories. We only measured the inter-lip distance for labial consonants because the Wisconsin x-ray database provides only one dimension of lip motion. Additionally, measuring time-to-voice for labial consonants is useful since they are often used in experiments investigating audiovisual speech perception [31],[32]. We estimated the time-to-voice for consonants either when they appeared at the beginning of a word such as “PROBLEM”, “BUT”, “FORM”, and “MAJOR” or produced in a vowel-consonant-vowel (VCV) context such as “ABA”, “APA”,“AMA” and “AFA”.

Consonants typically involve a rapid closing of the mouth followed by an opening for subsequent sounds. This consistent close-to-open state allows us to identify, across subjects, the time for such a gesture. Inspecting the velocity profiles along with the inter-lip distance profile allows one to identify the dynamics of the mouth preceding the closing gesture of a consonant. Therefore, we fixed the onset of the sound as the first landmark and then looked backward in time until the point where the inter-lip distance started to decrease. At this point, the velocity of the lower and upper lips should increase because the mouth is beginning to close. We then identified the second point as the point at which the lip velocity again changes direction; this gives us the point at which the mouth is half closed and the closing gesture is slowing down. The time in milliseconds between either of these two points and the onset of the sound provided us with estimates of the time-to-voice for each speaker. We aligned the time courses of lip markers to the sound onset to plot individual gestures and averages.

## Results

### Visual and auditory information in speech are robustly correlated

**Figure 2:**
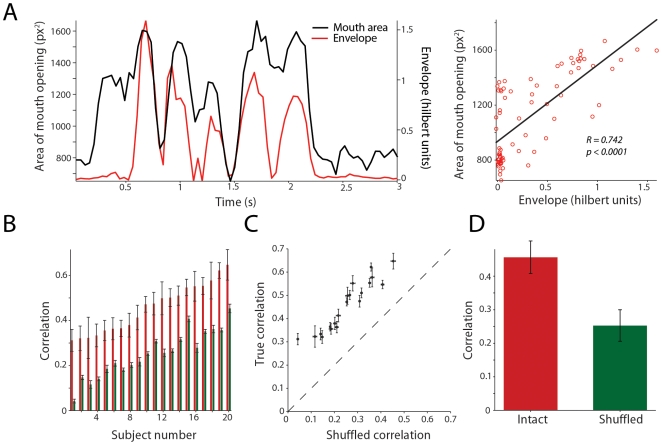
Average correlations between the area of the mouth opening and the audio envelope. A - Left, Area of the mouth opening and the auditory envelope for a single sentence from a single subject in the grid corpus as a function of time in seconds. X–axes depict time in seconds. Y–axes on the left depict the area of the mouth opening in pixel squared. Y-axes on the right depict the envelope in Hilbert units. Right, scatter plot of the envelope and area of the mouth opening along with the corresponding regression line. Each red circle denotes a single point in the speech time series. Black line denotes the linear regression between the area of the mouth opening and the envelope power. Correlation coefficient between auditory and visual components for this sentence was 0.742 (p<0.0001). B - Average rank ordered intact correlations (red bars) and shuffled correlations (green bars) for the 20 subjects analyzed in the GRID corpus. X-axes depict subject number; Y- axes depict the correlations. Intact correlations for each subject were the average across all sentences analyzed for that subject. Error bars denote standard error of the mean. Shuffled correlations were computed as an average correlation between all non paired auditory envelopes and the mouth area function for each subject. C - Scatter plot of the average intact versus the average shuffled correlations for the 20 subjects in the dataset. D - Mean intact and shuffled correlations over the 20 subjects in the GRID corpus. Error bars denote standard error of the mean.

Prior studies of audiovisual speech have suggested that the envelope of the auditory signal and the area of the mouth opening were correlated [13],[33]. However, such measurements were done on only a few sentences. Therefore, as our first comprehensive analysis of the statistics of audiovisual speech, we correlated the mouth area function with the wideband auditory envelope for several sentences from 20 different subjects from the GRID corpus. Figure 2A plots the wideband auditory envelope and the mouth area function (left panel) and a scatter plot of the two variables (right panel) for a single sentence spoken by a single subject. The auditory envelope was highly correlated with the mouth area function for this sentence (R = 0.742, p<0.0001).

Similar results were observed across the population of subjects (n = 20). The bar graph in Figure 2B plots the average rank-ordered correlation coefficient between the mouth area function and the wideband auditory envelope for the 20 subjects in our population. The average correlation across subjects between mouth area function and wideband envelope was 0.45(SD = 0.1) and the correlations ranged from 0.31 to 0.65. This suggests that there is a strong relationship between the temporal patterns of the mouth area function and the acoustic envelope, but significant intra- and inter-talker variability. To ensure that such correlations were not due to broad statistical relationships between the auditory and visual signals, we performed a shuffled correlation analysis. For a given subject, the mouth area function from one sentence was correlated with the auditory envelope of all the other sentences analyzed for that subject. This was repeated for all the sentences, providing a rigorous measure of the baseline level distribution of the correlations between auditory and visual information. Figure 2C plots the mean intact correlation versus the mean shuffled correlation. For every subject in our dataset, the mean intact correlation was significantly higher than the corresponding shuffled correlation (t (19) = 24.03, p<0.0001). Mean intact and shuffled correlations for the population are shown in Figure 2D.

**Figure 3:**
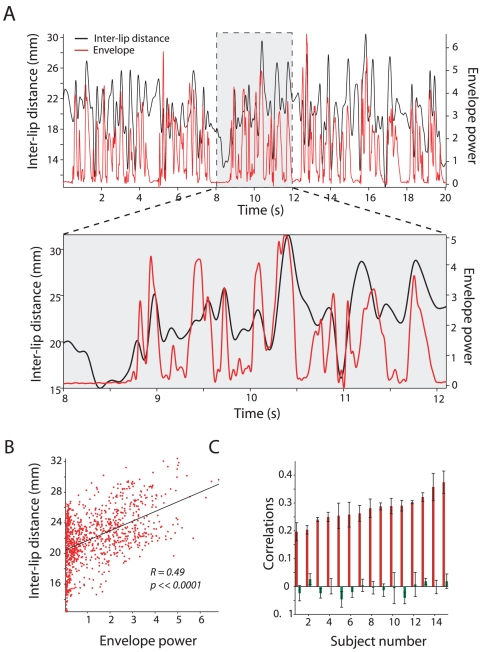
Average correlations between visual and auditory segments for longer speech materials. A - Top, Inter-lip distance and the auditory envelope for a single 20 second segment from a single subject in the X-ray database as a function of time. X –axes depict time in seconds. Y–axes on the left depict the distance between the lower and upper lip in millimeters. Y-axes on the right depict the power in the wideband envelope. Bottom, shows a zoomed in portion of the 8–12 second time segment of the same data shown in A. Clear correspondences are present between the inter-lip distance and the auditory envelope. B - Scatter plot of the envelope power and inter lip distance along with the corresponding regression line. Each red circle denotes a single point in the speech time series. Black line denotes the linear regression between the inter-lip distance and the envelope power. Correlation coefficient between auditory and visual components for this sentence was 0.49 (p<0.0001). C - Average rank ordered intact correlations (red bars) and shuffled correlations (green bars) for the 15 subjects analyzed in the Wisconsin×ray database. X-axes depict subject number; Y- axes depict the correlations. Intact correlations for each subject were the average across all speech segments analyzed for that subject. Error bars denote standard error of the mean. Shuffled correlations were computed as an average correlation between all non-paired auditory envelopes and the inter-lip distance for each subject.

To test whether these same auditory-visual correlations held true for longer speech streams (beyond just single, canonical sentences), we analyzed the correlations between the inter-lip distance and the auditory envelope for long passages of prose (durations spanning 16–25 seconds) from the x-ray database. Figure 3A plots the envelope and the inter-lip distance from a single subject speaking a 20-second passage. The bottom panel of the same figure shows an expanded view of the 8–12 second time segment. Consistent with the results from the GRID corpus, clear correspondences were observed between the visual signal and the auditory envelope. Figure 3B shows the scatter plot of the envelope power and the inter-lip distance. The correlation between the inter-lip distance and the envelope for this twenty-second segment was 0.49 (p<0.0001). Figure 3C plots the rank-ordered intact correlation along with the shuffled correlation for the 15 subjects in this dataset. The average intact correlation across subjects was 0.278 (SD = 0.05) and correlations ranged in magnitude from 0.197 to 0.375. Although small, the importance of such an effect is revealed when contrasted relative to the magnitude of the average shuffled correlations. The average shuffled correlations for the subjects was −0.0067(SD = 0.0293), ranging from −0.045 to 0.0265. Again, a paired t test across subjects revealed that the intact correlations were significantly different from the shuffled correlations (t (14) = 23.16, p<0.001).

One potential confound is that such correlations may be unique to the English language and would not be observed in other languages. We therefore analyzed the audiovisual correlations for spontaneous speech from two different French speakers in the same way as the GRID corpus by correlating the extracted inter-lip distance with the wideband envelope of the auditory signal. Mean intact correlation between the visual and auditory signals for the two speakers in this database were 0.22 and 0.29 and mean shuffled correlations were 0.01 and −0.01 respectively. The mean intact correlations were again significantly greater than the shuffled correlations for both speakers (speaker 1, t (727) = 8.1, p<0.0001, speaker 2, t (721) = 5.97, p<0.0001). Therefore, in three different databases and across two languages (English and French), visual information as indexed by either inter-lip distance or mouth area function were robustly correlated with the wideband envelope of the auditory signal. Such correlations were robust for both small sentences as well as longer, more extended passages.

### Relationship between the spectral structure of the voice and dynamics of the mouth

Having established that the wideband envelope of the auditory signal was robustly correlated with the area of the mouth opening, we next tested whether such correlations between the envelope and the visual signal varied as a function of the spectral frequency band of the acoustic signal. Since formant frequencies are important for perception of speech sounds such as vowels, correlations between formants and visual signals could provide important cues to articulatory dynamics especially in noisy auditory conditions.

**Figure 4:**
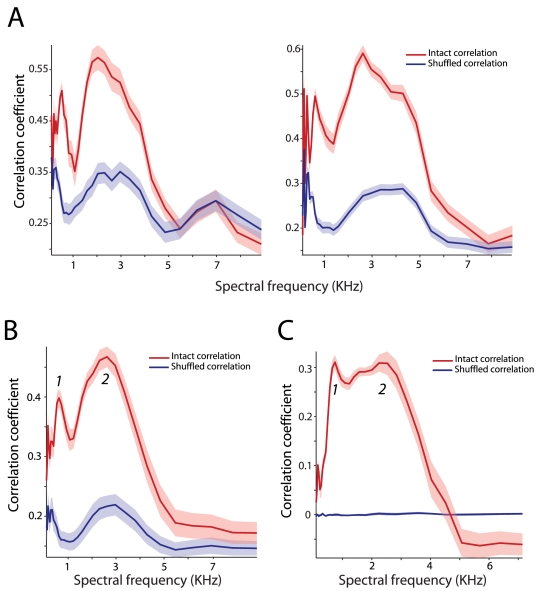
Correlation between visual and auditory components as a function of spectral frequency. A - Correlation coefficient between the area of the mouth opening and the envelope of each spectral band for two different subjects. X-axes depict the spectral frequency in KHz. Y-axes depict the correlation coefficient. The red line denotes the intact correlation between the area of the mouth opening and the auditory envelopes. Blue line denotes the average shuffled correlation for that subject. Shaded region denotes standard error of the mean. B - Average intact and shuffled correlation as a function of spectral frequency for the 20 subjects in the GRID corpus. Figure conventions are as in A. C - Average intact and shuffled correlation as a function of spectral frequency for the subjects from the Wisconsin X-ray database. Figure conventions as in A.

Figure 4A shows the intact and shuffled correlations between the mouth area function and the narrowband envelope as a function of spectral frequency for two different subjects from the GRID corpus. Two regions of high correlations separated by a region of relatively low correlation were observed. The first peak spanned a range 300–800 Hz and the second, larger peak, spanned a range from 1 to 3.5 KHz. This second 1–3.5 KHz region overlaps with the F2–F3 region of formant space. Across the population of subjects, the same pattern was found with correlations again peaking at ∼600 Hz and in the 3 KHz range (Figure 4B). In particular, the first peak was observed at 648 Hz (SD = 182 Hz) and the second peak at 2900 Hz (SD = 398 Hz). We observed no correlations for frequencies greater than 5 KHz.

The same analyses were conducted on the Wisconsin X-ray database. Figure 4C plots the intact and shuffled correlations between the inter-lip distance and the narrowband envelopes averaged across subjects. Two peaks were again observed in the correlation. The first peak was found at 720 Hz, and the second peak at 2.44 KHz. For the French audiovisual database, corresponding peaks in the correlation were observed at 530 Hz and 2.62 KHz. Thus, the results from all three databases suggest that the F1 region (300–800 Hz) and the F2–F3 region (∼3 KHz) are closely related to visual information.

### Temporal structure of audiovisual speech

**Figure 5:**
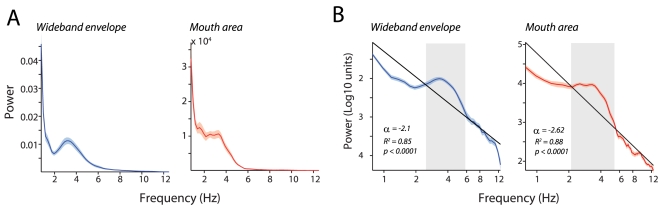
Temporal modulations in the visual and auditory signals. A - Left, average multitaper Fourier spectrum of the wideband auditory envelope across all sentences for a single subject from the GRID corpus. X-axes depicts frequency in Hz. Y-axes depicts power. Shaded regions denote the standard error of the mean. Note the peak between 2–7 Hz. Right, average multitaper Fourier spectrum of the mouth area function across all the sentences for the same subject shown in the left panel. X-axes depicts frequency in Hz. Y-axes depicts power. Shaded regions denote the standard error of the mean. B - Left, average multitaper Fourier spectrum of the wideband auditory envelope across all sentences for the same subject shown in A plotted on log-log axes. X-axes depicts frequency in Hz. Y-axes depicts power in log10 units. Shaded regions denote the standard error of the mean. Black line denotes a 1/f fit to the data. Deviations from 1/f are usually observed when there are rhythmic modulations in a signal. Right, average multitaper Fourier spectrum of the mouth area function across all the sentences for the same subject. Figure conventions as in the left panel.

**Figure 6:**
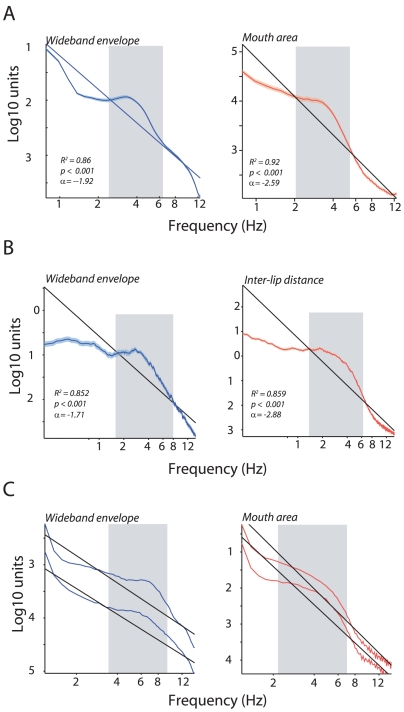
Temporal modulations in the population. A - Left, average multitaper Fourier spectrum of the wideband auditory envelope across all subjects in the GRID corpus plotted on log-log axes. Right, average multitaper Fourier spectrum of the mouth area function across all subjects in the GRID corpus plotted on a log-log axis. Figure conventions as in 5B. Gray shading denotes regions in the 2–7 Hz band, which seem to deviate from the 1/f fit. B - Left, average multitaper Fourier spectrum of the wideband auditory envelope across all subjects in the Wisconsin x-ray database plotted on log-log axes. Right, average multitaper Fourier spectrum of the inter-lip distance across all subjects in the Wisconsin x-ray database plotted on a log-log axis. Figure conventions as in 5B. C - Left, average multitaper Fourier spectrum of the wideband auditory envelope averaged over the entire spontaneous speech segment for the two subjects from the spontaneous speech database plotted on log-log axes. Right, average multitaper Fourier spectrum of the mouth area function for the two subjects in the spontaneous speech database plotted on a log-log axis. Figure conventions as in 5B.

The next analysis we performed was to analyze the temporal structure of the auditory and visual components of speech. We performed this for two reasons. First, as speech inherently evolves in time, no set of natural statistics of speech would be complete without investigating its temporal dimensions. Second, research suggests that the time-varying dimensions of visual speech are probably one of the most important information bearing dimensions [34] and so far there has been only a limited description of the temporal properties of visual speech [35]. We therefore computed the frequency spectra of the envelopes of the auditory signal *and* the corresponding mouth area function to identify the temporal structure of the auditory envelope and the movement of the mouth. Figure 5A shows the average spectrum of the audio envelope and the mouth area function for a single subject across 25 sentences. As expected, both the mouth area function and the audio envelope possessed a 1/f spectrum. Prominent peaks were observed between 2–7 Hz for both the mouth area function and the auditory signal suggesting that these modulations could perhaps drive the correlations we observed between visual and auditory signals. Figure 5B plots the spectrum of the wideband envelope and the mouth area function for the same data shown in Figure 5A on a log-log plot. In a logarithmic form, a 1/f function should have a linear relationship between logarithm of the power and the logarithm of frequency. The spectra were well fitted by a function of the form log Y = logA−α logf (R^2^ = 0.85, p<0.0001 for the auditory envelope; R^2^ = 0.88, p<0.0001 for the mouth area), but significant deviations from the 1/f fit were observed in the 2–7 Hz region for both the auditory envelope and the mouth area function. The alpha values were −2.1 for the auditory envelope and −2.62 for the mouth area function. We averaged these spectra across all 20 subjects for both the mouth area function and the sound and observed a similar pattern for the population. Figure 6A shows the average spectra across all 20 speakers in the GRID corpus for the audio envelope and the mouth area function. We also fitted the population average with the same function. The fitted value of α was −2.59 for the mouth area function and the corresponding value for the auditory envelope was −1.92.

Similar modulations were observed for the inter-lip distance and the auditory envelope from the x-ray database. Figure 6B plots the average spectrum of the auditory envelope and the inter-lip distance over all 15 subjects in the x-ray database. Again significant fits were observed (R^2^ = 0.85, p<0.0001) for both inter-lip distances and the envelope. Consistent with the results from the GRID corpus, modulations were found in the 2–7 Hz frequency range. The fitted value of α was −1.71 for the audio envelope and −2.88 for the inter-lip distance. Finally, Figure 6C shows similar spectra for the auditory envelope and the mouth area function for the two speakers in the French audiovisual database. Our results therefore suggest that some of the relevant information in audiovisual speech indexed by the envelope of the sound and the mouth area function is present in the 2–7 Hz temporal frequency band.

### Coherence between auditory and visual signals

**Figure 7:**
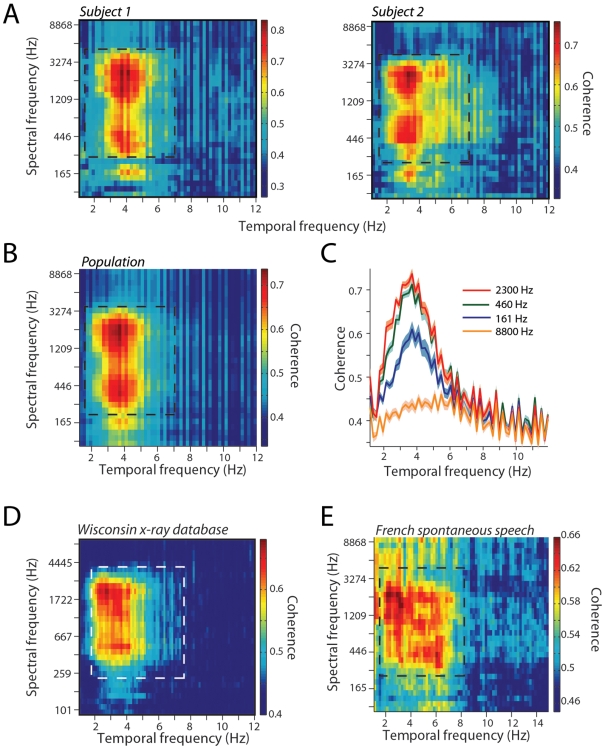
Coherence between vision and audition. A - Left, heat map shows the coherence between the mouth area function and the auditory signal as a function of both spectral frequency band and temporal modulation frequency for a single subject from the GRID corpus. X-axes depicts temporal modulation frequency in Hz. Y-axes depict the spectral frequency in KHz. Square drawn in dashed lines depicts the region of maximal coherence between the visual and auditory signals. Right, heat map for another subject from the GRID corpus. Figure conventions as in the left panel. B - Average coherence between the mouth area function and the auditory signal as a function of both spectral frequency band and temporal modulation frequency for the twenty subjects in the GRID corpus. Figure conventions as in the left panel of A. C - Average coherence between the mouth area function and the auditory signal for four different spectral frequencies (8.8 KHz – orange, 2.3 KHz – red, 161 Hz – blue, 460 Hz – green) across all subjects in the GRID corpus as a function of temporal frequency. Shaded regions denote the standard error of the mean. D - Average coherence between the inter-lip distance and the wideband auditory envelope as a function of both spectral frequency band and temporal modulation frequency for the fifteen subjects in the Wisconsin x-ray database. Figure conventions as in A. E - Average coherence between the area of the mouth opening and the wideband auditory envelope as a function of both spectral frequency band and temporal modulation frequency averaged across the two subjects from the French spontaneous database. Figure conventions as in A.

Thus far, our analyses revealed three important results. First, auditory, and visual signals were highly correlated with each other. Second, such correlations were maximal in the F2–F3 region of formant space. Third, both visual signals and auditory signals were modulated in a 2–7 Hz frequency band. We next examined whether there was a relationship between the temporal modulations of audiovisual speech and the formant space. To do this, we adapted a coherence measure to analyze the relationship between the visual and auditory signals as follows. For each sentence, we took the narrowband envelopes and the mouth area function and then computed the coherence as a function of the temporal modulation frequency. This provided us with an estimate of the coherence for each spectral frequency band. We then averaged this coherence estimate across all sentences for each subject and then subsequently across subjects. Figure 7A (left panel) shows the coherence as a function of both spectral and temporal frequency for a single subject from the GRID corpus. A clear region spanning 2–7 Hz was observed for multiple spectral frequency regions. Consistent with our prior results, coherence was maximal for the F2–F3 regions and robust for the F1 region. The right panel shows similar coherence estimates from another subject. Figure 7B shows the average coherence across all 20 subjects in the GRID corpus database. Maximal coherence is observed in three regions one centered at ∼165 Hz, the second at ∼450 Hz and the third the F2–F3 formant region between 1–3 KHz. To illustrate this further, Figure 7C shows coherence as a function of temporal frequency for a selected narrowband in these different frequency band ranges — 161 Hz, 460 Hz and 2300 Hz in comparison to the 8800 Hz frequency band. Maximal relationships are observed in the 300 to 800 Hz frequency band and in the 1–3 KHz bands with slightly smaller coherence also present in the 165 Hz frequency bands. Such coherence appears maximal in the 2–7 Hz temporal frequency bands.

Results from the two other databases closely followed the pattern found for the GRID corpus. Figure 7D shows the coherence between the auditory envelope and the inter-lip distance for the Wisconsin x-ray database. The coherence computations in this case were performed on 6-second segments shifted by 5-second increments to ensure that coherence was computed on similar time scales across all three databases. Again, coherence was maximal in the 2–7 Hz region. Figure 7E shows the average coherence between the auditory envelope and the mouth area function for the French spontaneous audiovisual speech database with very similar results. Since there were only two subjects, estimates of coherence are noisier, but are observed again in the 2–7 Hz temporal frequency region and for the two spectral frequency ranges of F1 and F2–F3. Thus, auditory signals as represented by envelopes of different spectral frequencies and the visual signals as represented by the mouth area function or the inter-lip distance are closely related to one another and are together modulated in a 2–7 Hz temporal frequency region.

### Mouth movements lead the voice within a small temporal window

One characteristic feature of speech is that the onset of visual signals usually precedes the onset of the sound. This can for example be seen in Figure 2, where the area of the mouth opening increases a few hundred milliseconds prior to the onset of the sound. Other researchers have noted this and shown (or suggested) that the onset of visible articulatory motion in the face before the onset of the mouth may be important for the processing of speech [12],[31],[36]. Yet, there has been no systematic measurement of the delays between the onset of such facial motion and the onset of the voice. We therefore used the high temporal resolution of the Wisconsin x-ray database to identify the dynamics of lip movements before the onset of the voice. Since this database only provides 1-dimensional measures of the lip motion, we only analyzed the labial consonants such as (/p/, /b/, /m/) and the labiodental consonant (/f/). Such bilabial consonants are often used in studies of audiovisual speech perception [31],[37].

**Figure 8:**
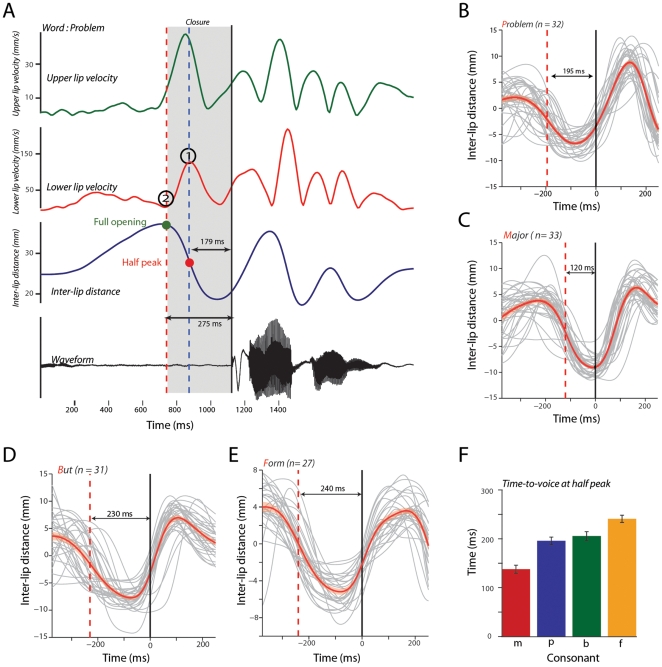
Measuring time-to-voice for consonants at the beginning of words. A - Visual and auditory dynamics during the production of the word “PROBLEM” by a single speaker. Green and red lines denote the velocity profiles of the upper and lower lips respectively. Dark blue line denotes the inter-lip distance as a function of time. The waveform of the sound is shown in black. Blue dashed line denotes the point of maximal lower lip velocity (marked by (1)). Red dashed line denotes the point of zero lower lip velocity (marked by (2)). Solid black line denotes the onset of the sound. The greens dot denotes the maximal mouth opening before onset of the sound. The red dot denotes the half peak point of the mouth opening. X-axes depict time in milliseconds. B - Inter-lip distance for different subjects and average as a function of time for the bilabial plosive /p/ aligned to the onset of the sound. Red dashed line denotes the half peak opening of the mouth. X-axes depict time in milliseconds, y-axes depict inter-lip distance in mm. Solid dark line denotes the onset of the sound. Gray lines denote traces from individual subjects. Red line denotes the average across subjects. Shaded regions denote the standard error of the mean. C - Inter-lip distance for different subjects and average as a function of time for the bilabial consonant /m/ aligned to the onset of the sound. Figure conventions as in B. D - Inter-lip distance for different subjects and average as a function of time for the bilabial plosive /b/ aligned to the onset of the sound. Figure conventions as in B. E - Inter-lip distance for different subjects and average as a function of time for the labiodental /f/ aligned to the onset of the sound. Figure conventions as in B. F - Average time to half opening from the onset of the sound for the three bilabial consonants and the labiodental across subjects. Error bars denote the standard error of the mean.

A description of the analysis we used to estimate the time-to-voice for bilabial consonants is as follows. The production of consonants involves a transition from an open to a closed mouth state. Our reference was the onset of the sound, which we estimated from the speech waveform. We then looked back in time until we found the point where the inter-lip distance began to decrease. This is the point at which the mouth begins to close. To estimate the point at which the inter-lip distance decreases one can use the derivative, which in our case is the velocity of the lips. This is marked by the point (2) and a red dotted line in Figure 8A and marks the beginning of the closing gesture. We next identified the point at which the mouth is half open because eye movement data suggest that both monkeys and humans fixate on the mouth at the onset of mouth movements, not necessarily when it is fully open [38],[39]. At the half-open point, the lips are no longer accelerating towards one another, and at this point, there should be a reversal in the velocity of the lips. This is marked by the point (1) and a red dot on the inter-lip distance on the lower lip velocity profile (Figure 8A). The time between the “half-open” point and the onset of the sound provides an estimate of the time-to-voice.

Figure 8A plots the velocities of the upper and lower lip along with the inter-lip distance and sound for the word “PROBLEM” which starts with the bilabial consonant /p/. As the velocity profiles reveal, the upper and lower lip seem to have very similar kinematics. As is characteristic of bilabial plosives, the mouth opens and rapidly closes until just before the release of the sound. To estimate the time-to-voice (the delay between the onset of the mouth movement and the sound), we took the point of zero (marked by ‘2’) in Figure 8A) and maximum lower lip velocity (marked by ‘1’) as two landmarks. As the dotted lines show, the zero velocity point corresponds to the point where the mouth is maximally open before beginning the closing motif and the maximum velocity point corresponds to the half “maximum” of this closing action. The time between each of these points and the time of sound onset provides an estimate of the “time-to-voice”. In this particular example, the estimated time-to-voice at the half-open point was 179 milliseconds. Figure 8B shows the mean subtracted inter-lip distance for the word PROBLEM aligned to the onset of the sound for the 32 speakers analyzed from the database. The grey lines denote the traces from individual subjects with the means subtracted. Figures 8C, 8D and 8E show corresponding plots for the words “MAJOR”, “BUT” and “FORM” which correspond to the two other bilabial plosives, /b/ and /m/, and the labiodental, /f/. Figure 8F shows the mean time-to-voice (mean range = 137–240 ms) for the four consonants across all speakers in the database (mean±sd: /p/, 195±60 ms (n = 32 subjects); /m/, 137±64 ms (n = 33 subjects); /b/, 205±69 ms (n = 31 subjects); /f/, 240±38 ms (n = 27 subjects)).

**Figure 9:**
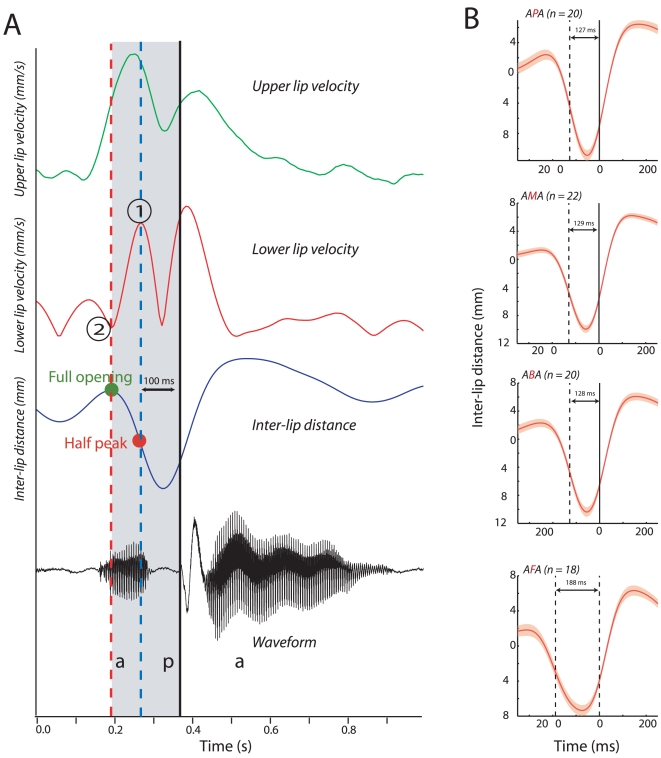
Context modifies time-to-voice for consonants. A - Visual and auditory dynamics during the production of the word “APA” by a single speaker. Conventions as in Figure 8A. B - Average inter-lip distance as a function of time for the word APA, AMA, ABA and AFA across all subjects in the database aligned to the onset of the respective consonants (/p/, /m/, /b/, /f/). X-axes depict time in milliseconds, y-axes depict the mean corrected inter-lip distance in mm. Shaded regions denote the standard error of the mean.

We next analyzed whether the time-to-voice varies with the context in which the consonant sounds are produced. That is, does the timing of lip movements vary when the mouth goes from open-to-close-to-open states? We estimated the lip kinematics for the same set of consonants when they are produced in a vowel–consonant–vowel (VCV) syllable to identify how context modifies the lip closure dynamics. Figure 9A shows the velocity profiles for the lower and upper lips as a function of time, for the word APA produced by a single speaker. The time from half-open state is 100 ms for this example, which is different from the 179 ms we observed when the consonant /p/ was at the beginning of a word. Figures 9B shows the inter-lip distance traces for the words APA, AMA, ABA and AFA aligned to the sound onset. The mean range for these VCV syllables was 127 to 188 ms. For each VCV utterance independently: /p/, 127±21 ms (n = 20 subjects); /m/, 129±19 ms (n = 22 subjects); /b/, 128±20 ms (n = 20 subjects); /f/, 188±29 ms (n = 18 subjects). These data suggest that, even in a different articulatory context, the time-to-voice variable is within a time window of 100–300 ms, though it is clear that movement is faster than when that consonant is at the beginning of a word.

## Discussion

We characterized the natural statistics of audiovisual speech. We found that there were robust correlations and close temporal correspondences between the area of the mouth opening and the auditory envelope, and that the strongest correlations occurred in two frequency bands–below 1 kHz and between 2 and 3 kHz. Across two languages and contexts, both mouth movements and the acoustic envelope were temporally modulated in the 2–7 Hz frequency range. The timing of mouth movements relative to the onset of the voice was consistently between 100 and 300 ms. We will discuss the implications of each of these audiovisual speech statistics in turn.

### Visual and auditory components of speech are coherent

The time course of opening and closing of the mouth is tightly locked to the acoustic envelope. Does close temporal correspondence between vision and audition offer any advantage for speech perception or is it just an incidental consequence of the speech production mechanism? Psychophysical results suggest that the close temporal correspondence between faces and voices mediates at least some of the observed behavioral effects in audiovisual speech perception. For example, reversing or shifting the auditory components of sentences in time leads to a loss of multisensory advantages [40] and speech detection in noise is enhanced for matched compared to unmatched audiovisual sentences [33]. Indeed, research suggests that the time-varying dimensions of visual speech are probably one of the most important information bearing dimensions in speech [34]. For example, experiments with point light-displays, where the dynamic face is replaced by 12–20 points which mimic its movement, reveal that such purely dynamic information can enhance speech perception [41] and can even be used to induce McGurk effects (albeit at a reduced level) [42]. Similarly, spatial low pass filtering of the moving face while keeping temporal characteristics intact preserves the audiovisual gain for understanding spoken sentences [43]. Computationally, audiovisual coherence in speech has been exploited to separate out an acoustic speech signal from other acoustic signals [44], and temporal synchrony is a key parameter used to segregate audiovisual sources, especially speech sources [45].

### Mouth movements are linked to spectral peaks

Macroscopic relationships between the state of the mouth and the amplitude of the sound have always been known. For example, sound amplitude is generally larger when the mouth is opened compared to when it is closed. However, beyond such simple relationships, our data show that the dynamics of the opening and closing of the mouth can be linked to specific frequency bands in the acoustic speech signal: they correlate well with spectral regions corresponding to formant frequencies. This observation is well supported by previous attempts to measure the correlation between visual and auditory components of speech. Grant and Seitz [33], using methods similar to ours, reported robust correlations between the F2–F3 component and the mouth area for three sentences from a single speaker. This relationship between the opening and closing of the mouth and the spectral structure of the sound is somewhat surprising given that the production of speech requires a precise orchestration of the activity of several different components of the vocal tract anatomy. Besides the mouth and the lips, which form the most visible parts speech production process, complex adjustments of the pharynx, tongue and jaw are also required to actually produce a speech utterance [46],[47].

It is important to note that our measurements only take into account one feature of the available visual signals from a dynamic face during speech. Specifically, we concentrated on the spreading motion of the mouth since we could measure that in a straightforward manner from the videos found in these databases and with the existing computer vision algorithms. However, facial regions beyond the mouth are also linked to the dynamics of the vocal tract and thus to speech acoustics. In a series of studies, Munhall, Vatikiotis-Bateson and collaborators used three dimensional kinematic tracking of facial and interior articulatory movements during speech and analyses of the corresponding acoustic signal to study the relationship between the two modalities [35], [48]–[50]. High correlations were observed between the visual markers and the acoustic dimensions such as RMS amplitude and spectral structure. Even head movements can provide informative visual cues [49],[50]. Indeed, the multitude of visual cues can provide information about not only what is being said, but also who is saying it [51],[52]. Finally, we did not measure the dynamics of structures such as the tip of the tongue and its placement relative to the teeth [53]; this relationship can also be a visual marker of speech production. Further studies of the dynamics of these structures are needed to establish the role they may play in audiovisual speech and whether their dynamics are different from the ones we observed for the opening and closing of the mouth. That said, it should be noted that movements of the tongue–an articulator not necessarily coupled with the face–can be well-estimated just by using facial motion; it frequently displays the same temporal pattern as the mouth during speech [48].

What advantage does this close relationship between spectral structure and the opening and closing of the mouth provide? On the perceiver's side, estimating the articulatory goals of the producer is probably useful for robust speech perception, since it allows one to make better inferences about the auditory signal. It is therefore fortuitous that the movements of the mouth can actually provide information about the spectral content of the auditory signal. This suggests that the brain can use the kinematics of the face to access the articulatory processes involved in the production of the acoustic signal and thereby refine its interpretation of the acoustic signal.

One attractive hypothesis is that audiovisual speech perception is mediated, at least in part, by “peak listening”, which suggests that peaks in the visual signal (e.g. mouth opening), provide cues to the peaks in the auditory signal (e.g. formant peaks) [40]. Our data suggest an extension of the peak listening theory: the mouth opening and closing may not only enhance perception at the peaks, but also track the auditory envelope more generally and is thus a robust, *continuous* cue. The importance of a continuous visual signal is suggested by the extensive psychophysical data on speech perception. These data point to the critical contribution of the acoustic envelope for intelligibility [23],[24],[54],[55]. Further support comes from MEG/EEG studies which show that sentence comprehension correlates with how well the auditory cortex can track the acoustic envelope [56]–[58]. Close temporal correspondence between mouth area function and the acoustic envelope could provide an important boost during the information rich regions of the auditory signal [12].

### Slow temporal modulations in audiovisual speech

Our statistical analyses reveal that temporal modulations in speech are about 2–7 Hz for both vision and audition. These measurements concur with prior reports of the temporal dynamics of speech. Ohala [59] measured the intervals between successive jaw movements during reading and estimated a rate of approximately 5 Hz as the underlying time scale for speech. Munhall and Vatikiotis-Bateson [35] used infrared emitting diodes to measure the movements of the face and estimated temporal modulations in visual speech to be in the range 0–10 Hz. This range of temporal modulations is important for audiovisual speech perception. For example, reduction of the frame rate of visual speech removes the audiovisual benefit [7],[60],[61]. Similarly, audiovisual advantages disappear for synthetic talkers speaking at rates faster than normal speech [40]. These studies along with the corresponding studies of auditory-only speech [24],[54],[55] suggest that slow temporal modulations in the 2–7 Hz range *across modalities* are indeed important for the perception of speech.

### Consistency with amodal accounts of speech perception

Our analyses of the natural statistics of audiovisual speech show similar temporal modulations for the visual and auditory signals and reveal that the area of the mouth opening is closely related to formant frequencies. These results suggest that, at multiple levels, there are several redundant cues which all point to a description of the speech gesture. Taken together, they indicate that the speech signal is, to some degree, agnostic with regard to the precise modality in which it is perceived. These observations correspond to the tenets of an amodal, or modality-neutral, description of speech [15],[53]. According to this viewpoint, visual and acoustic signals are shaped in the same way by a speech gesture from a signaler. Integration is therefore a consequence and property of the information in the input. By this it is meant that speech perception does not involve a series of complex transformations in each modality independently followed by a mechanism to bind this information. On the contrary, auditory and visual components share several redundancies and the process of speech perception involves the extraction of these redundancies. Our measured parameters are consistent with an amodal description of a speech gesture. As formant frequencies are predicted by area of the mouth opening and vice versa, they point to a modality-free description of the vocal tract. Similarly, both the acoustic envelope and the area of the mouth opening share a common temporal axis of 2–7 Hz. This common modulation has been previously suggested as a possible amodal metric of speech [15],[53].

The suggestion that speech (and other vocal communication systems) could be processed in an amodal fashion is also attractive from a neurophysiological perspective [62]. Research suggests that most, if not all, neocortical areas are multisensory, including areas such as primary visual and auditory cortex, which have long been considered the foundation of cortical unimodal processing [63]. This strongly suggests that both auditory and visual components of speech are processed together at the earliest level possible in neural circuitry.

### Relationship between neocortical oscillations and the slow modulations in audiovisual speech

Recent theories of speech suggest that the temporal modulations in speech are well matched to two key brain rhythms in the same frequency range [11],[12]. In an expanded form, these theories state that the importance of the 2–7 Hz temporal frequency modulations may be due to it being in the same range as two very important neocortical rhythms — the delta (1–4 Hz) and theta (4–8 Hz) band.

Studies have shown that oscillations at different amplitudes seem to be phase amplitude coupled in a hierarchical fashion [64],[65]. For example, this means that amplitude of a 25–50 Hz (putative “gamma”) frequency band is modulated according to the phase of an underlying 5–9 Hz oscillation. The amplitude of this 5–9 Hz (putative ‘theta’) oscillation is in turn controlled by the phase of a slower 1–2 Hz (usually termed ‘delta’) oscillation. The activity in the lowest frequency bands in the auditory cortex are known to be entrained by rhythmic stimulation [64]. Our finding that audiovisual speech modulations in the 2–7 Hz range could be such a rhythmic input, entraining oscillations in the delta and theta bands. In other words, the activity of circuits in auditory cortex in the delta and theta band would be modulated by the slow modulations in audiovisual speech.

In addition to this entrainment of slow brain rhythms to speech modulations, a provocative theory of speech, termed the “sampling-in-time”, suggests that auditory-only speech is preferentially processed in two time windows [11]. The first time window is in the timescale of a stressed syllable [14] or ∼150 to 300 ms and is mediated by the theta (3–8 Hz) rhythm endogenous to the auditory cortex [64],[66]. The second window is between 20–40 ms and thought to process formant transitions and probably mediated by the gamma rhythm. Evidence for this first, slower time window, is considerable. For example, observers who listen repeatedly to sentences where local segments of varying durations are time-reversed have few difficulties recognizing the content of the sentence, but only when the reversed segment durations are less than 100 ms [67]. When segment durations exceed 130 ms (i.e., disruptions that fall across the theta range), intelligibility drops to chance levels. Similarly, when speech rates are faster than 8 Hz, auditory cortical activity cannot track its modulations and there is a corresponding loss of intelligibility [56]. Finally, recent experiments reveal that the phase of theta band (3–8 Hz) activity in auditory cortex actively tracks the intelligibility of sentences [58].

Our data suggest that the sampling-in-time theory could be extended to audiovisual speech. According to our data, visual speech could assist in the segmentation and processing of sounds especially at the syllabic level. Since mouth movements are in a similar syllabic time scale, they could be useful in assisting in such chunking, perhaps replacing a modality when the signal from the other modality is ambiguous. Open and close states of the mouth could provide important cues to start and end points of syllables. Vision might also lead to phase resetting in auditory cortex, perhaps at the start of each syllable, thereby priming the auditory cortex to be maximally sensitive to auditory inputs it encounters subsequently [12], an issue which we explore in the next section.

### Visual cues precede auditory cues in natural audiovisual speech

The last observation from our measured statistics is that the time-to-voice (the delay between onset of mouth movements and the onset of the voice) is between 100 and 300 milliseconds. This was true for words with bilabial consonants at the beginning, as well for VCV sounds where the consonant (and thus the mouth closure) is embedded. Therefore, under naturalistic conditions, structures mediating audiovisual speech perception must be tolerant to delays between visual and auditory signals. This observation is consistent with behavioral studies of audiovisual speech. Auditory speech must lag behind matching visual speech by greater than 250 milliseconds before any asynchrony is perceived by human subjects [68]. In stark contrast, detecting audio-visual asynchrony in artificial stimuli requires timing differences of as little as 20 milliseconds [69]. Similarly, the McGurk illusion is robust with vision leading audition by up to 240 milliseconds [32],[70]. Thus, the time-to-voice range (100–300 ms) that we find for audiovisual speech is consistent with the sensitivity of human subjects: if the auditory component lags the visual component with delays beyond this range, then speech perception is disrupted. Furthermore, this time interval is in accord with the electrophysiological constraints for auditory versus visual latencies in brain structures involved in speech perception [12].

Schroeder et al [12] hypothesized that the onset of mouth motion prior to the voice could also lead to more complex network dynamics such as phase resetting and that subsequent auditory signals falling on high excitability peaks of this reset oscillation will lead to the amplification of speech perception. This idea is borne out of a study of multisensory responses in the primary auditory cortex, where it was observed that somatosensory stimuli, which are generally ineffective at eliciting supra-threshold responses in auditory cortex, nevertheless reset the phase of ongoing oscillations in auditory cortex. Auditory stimuli which subsequently arrived at the low excitability phase of this reset oscillation were suppressed, while responses to stimuli which arrived at the high excitability phase were enhanced [26]. A similar pattern of results has also been reported for visual-auditory interactions [71]. Some evidence for this process also comes from an EEG study of audiovisual speech which showed a latency facilitation for audiovisual speech compared to auditory alone speech in auditory regions which depended on the degree to which visual signals predicted the auditory signal. [31]. However, it is unclear how continuous mouth motion seen during the production of a sentence would modify such processing in primary auditory cortex or how shifting eye movements of observers [38],[39],[72] would modify the phase resetting of ongoing oscillations [73].

Here is perhaps a more plausible scenario that builds on Schroeder et al.'s [12] framework for the visual amplification of speech but that incorporates eye movements and facial dynamics. A dynamic, vocalizing face is a complex sequence of sensory events, but one that elicits fairly stereotypical eye movements: we and other primates fixate on the eyes but then saccade to mouth when it moves before saccading back to the eyes [38],[39]. Eye position influences single neuron and local field potential activity in multiple regions of auditory cortex [74],[75]. Therefore, one possibility is that the *eye fixations* at the onset of mouth movements send a signal to the auditory cortex which resets the phase of an on-going oscillation. Such effects have been for example already seen in the primary visual cortex [76]. This proprioceptive signal thus primes the auditory cortex to amplify or suppress (depending on the timing) the neural response to a subsequent auditory signal originating from the mouth. Given that mouth movements precede the voiced components of both human [77]and monkey vocalizations [25],[30], the temporal order of visual to proprioceptive to auditory signals is consistent with this idea. This hypothesis is also supported (though indirectly) by the finding that the sign of face/voice integration in the auditory cortex and the STS is influenced by the timing of mouth movements relative to the onset of the voice [25],[30].

One cautionary note is that our analysis of the time-to-voice focused only on bilabial consonants. Naturally, there is a far greater range of articulatory contexts which need to be explored. We focused on bilabial consonants because our X-ray database provided only a one-dimensional measure of mouth opening. Some prior work has attempted to measure the range of lip and jaw movements for a larger range of vowels and consonants, but only with a few subjects; estimates from this study were similar to ours [78]. Future work using the methods outlined in [78] or using the chroma key system [22] could be used to measure the time-to-voice for a larger range of articulatory contexts.

### The embodiment of speech

Our analysis of the natural statistics of speech suggests that the statistical structure of audiovisual speech might be well adapted to the circuits which are involved in its processing. This fits well with current ideas about the emergent nature of communication and behavior — that is they emerge via the interactions between the brain, the body and the environment [4],[5],[79]. Key features of this ‘embodiment’ approach are three-fold. First, our experience is multimodal. This is certainly true for speech [6]. Second, multiple overlapping and time-locked sensory systems enable the agent to act in the world [5]. The revelation that most, if not all, of the neocortex speaks to this claim generally [63], and the interactions between visual and auditory cortical regions which mediate face/voice integration speaks to vocal communication specifically [80],[81]. Finally, sensory and motor systems will be coupled so that stable features of the brain, body, and/or environment can be exploited to simplify perception and action [2],[4],[5]. It is this last principle that the data presented in this current study address. The statistical structure of audiovisual speech is such that it seems well matched to the circuits involved in processing it. First, modulations in the 2–7 Hz temporal frequency region overlap with the temporal sensitivity of neurons in the primary auditory cortex and, to some extent, the oscillatory structure of cortical networks more generally [82]. Second, the relative delay between visual and auditory inputs seems well matched to the relative latencies and processing delays for the respective modalities thereby ensuring that visual speech can help in the efficient processing of auditory speech.
